# COVID-19 vaccine uptake and barriers among Indigenous language speakers in Mexico: Results from a nationally representative survey

**DOI:** 10.1371/journal.pgph.0002921

**Published:** 2024-03-28

**Authors:** Lucía Abascal Miguel, Cesar A. Mendez-Lizarraga, Elizabeth M. Rojo, Jaime Sepúlveda

**Affiliations:** 1 Institute for Global Health Sciences, University of California San Francisco, San Francisco, California, United States of America; 2 Department of Population Health Sciences, Duke University, Durham, North Carolina, United States of America; Nanyang Technological University, SINGAPORE

## Abstract

Mexico faced a significant burden from the COVID-19 pandemic. Since the pandemic’s onset in 2020, numerous studies have underscored the substantial risk of COVID-19 death among Indigenous individuals. This study aims to assess COVID-19 vaccine uptake among Indigenous language speakers in Mexico, focusing on understanding the barriers they face in obtaining access to vaccines. We used Encuesta Nacional de Salud y Nutrición Continua (ENSANUT) 2022, a nationally representative health survey in Mexico to analyze data on self-reported COVID-19 vaccine status, reasons for not getting vaccinated, and other relevant covariates. We employed logistic regression to estimate odds ratios (ORs) for vaccine uptake and uptake barriers, all models were adjusted for potential confounders. Among 34,051 participants, 1793 individuals (5.23%) reported speaking an Indigenous language. Indigenous language speakers were found to have a lower vaccination rate (63%) compared to non-Indigenous language speakers (81%) (p <0.005). They were also 59% less likely to be vaccinated against COVID-19 (OR 0.41, 95% CI 0.27–0.62), even when adjusted for confounders. Among unvaccinated individuals, Indigenous language speakers were more likely to cite negative beliefs about the vaccine or fear as reasons for not being vaccinated (OR 1.82, 95% CI 1.11–3.00) while being less likely to report access barriers (OR 0.62, CI 95% 0.42–0.91). This study highlights disparities in COVID-19 vaccine uptake among Indigenous language speakers in Mexico. The findings underscore the urgent need for targeted, culturally appropriate public health interventions and the consideration of social and ethnic vulnerability in prioritizing vaccinations.

## Introduction

As of July 26, 2023, Mexico has reported 7,633,355 confirmed COVID-19 cases, resulting in 334,336 deaths, a gross underestimate given the lack of testing [1, 2]. The country experienced one of the highest mortality rates worldwide, with 2,622 cumulative deaths per million people, three times the global average of 872 deaths per million people [3]. Mexico also experienced the fourth highest total excess deaths globally, with 798,000 excess deaths in the first 2 years of the pandemic [4]. Furthermore, the pandemic has revealed significant disparities in its impact, disproportionately affecting marginalized communities and Indigenous populations in Mexico [5].

In 2022, out of 126 million Mexicans, around 19% self-identified as Indigenous, of those, one third or around 7.3 million speak an Indigenous language. Of those who speak an Indigenous language, only 12% or around 900,000 don’t speak Spanish [6]. This Indigenous population represents the most socio-economically disadvantaged group in the country [7]. Notably, 69.5% of Mexico’s Indigenous population, equivalent to around 8.5 million individuals, lives in poverty, with 28% living in extreme poverty [8]. Seventy-five percent of Indigenous people are concentrated in 8 states with Oaxaca and Chiapas, states in southern Mexico, having the highest concentrations [9]. The indigenous population in Mexico is very diverse, comprising 68 different pueblos originarios or Indigenous Peoples, each speaking a distinct language [6]. Continued poverty, racism and a history of structural inequalities compound the challenges for these communities, including limited incomes, restricted access to education and healthcare services, and high levels of food insecurity [8, 9].

In an effort to overcome these barriers, Mexican legislation has included healthcare provisions since 1991 with the ’Modelo para la Atención Intercultural a la Salud de los Pueblos Indígenas y Afromexicanos’ (Model for Intercultural Health Care for Indigenous and Afro-Mexican Peoples), which is aligned with the ’Ley General de Salud’ (General Health Law), the ’Plan Nacional de Desarrollo 2019–2024’ (National Development Plan 2019–2024), the ’Plan Sectorial de Salud 2019–2024’ (Health Sector Plan 2019–2024), and binding international agreements such as the ILO Convention 169 [10, 11]. These frameworks and laws advocate for the specific health needs of Indigenous and Afro-Mexican populations, promoting timely and efficient healthcare services, cultural competence among providers, dignified care, and the empowerment of these communities through the recognition and enforcement of their healthcare rights and autonomy [11].

The COVID-19 pandemic has further intensified the hardships faced by Indigenous populations. Discrimination also contributes to the complex situation, making it more challenging for these communities to cope with the pandemic’s impacts [12, 13]. Moreover, Indigenous communities often encounter multiple barriers due to their economic and social conditions, resulting in isolation and marginalization, which leads to limited access to healthcare services [14]. In 2018, only 18.5% of one-year-old children had full vaccination coverage. The coverage for Indigenous populations was up to 30 percentage points lower than the non-Indigenous population for vaccines such as BCG, Hepatitis B, HPV, Measles and Rubella, and Tetanus [15].

Globally, racial and ethnic minorities, including Indigenous communities, have faced a disproportionate burden during the COVID-19 pandemic, grappling with higher mortality rates and limited healthcare access [16]. In Mexico, numerous studies since the pandemic’s onset in 2020 have underscored the significant risk of COVID-19 death among Indigenous language speakers. These communities tend to be older and have higher comorbidity rates, leading to worse COVID-19 outcomes [17, 18]. Factors like marginalization and comorbidities further elevate the risk of COVID-19 fatality among Indigenous individuals [19]. Additional studies have revealed that Indigenous people receiving outpatient management experienced a higher fatality rate than non-Indigenous outpatients [14, 18, 20]. These communities also face delays in seeking care and have lower survival probabilities [21]. National statistics reveal that Indigenous individuals experienced an almost twofold higher mortality rate compared to non-Indigenous counterparts between 2020 and 2022 [22, 23]. The higher COVID-19 mortality rate among Indigenous populations is consistently observed across different regions of the country and at the population level, as municipalities with a higher proportion of Indigenous residents face increased mortality risks [5, 23].

COVID-19 vaccines have played a crucial role in reducing COVID morbidity and mortality worldwide since their rollout, preventing an estimated 14.4 million deaths globally within the first year of availability, with this number rising to nearly 20 million when using excess mortality data estimates [24]. However, the unequal distribution of vaccines can lead to significant disparities in access and health outcomes, particularly for marginalized communities [25]. Mexico’s COVID-19 vaccination campaign began in December 2020, prioritizing healthcare workers and seniors over 60, with the broader adult population following from May to July 2021. The policy acknowledged the need to vaccinate specific groups like Indigenous populations but lacked detailed plans for age-specific strategies within these groups [26]. In 2023, 75% of the adult population had received at least one dose of a COVID-19 vaccine [1]. Although there is no official data available regarding vaccinations categorized by ethnicity, a study based on the 2022 National Health and Nutrition Survey (Encuesta Nacional de Salud y Nutrición Continua 2022 (ENSANUT)) unveiled a decline in vaccination rates among individuals with the lowest welfare index and those residing in rural areas [27]. Other reports have shown that Indigenous communities experienced barriers in accessing vaccines and that vaccine hesitancy was present in these communities [28, 29]. Vaccine uptake among Indigenous populations was influenced by various factors, including limited access to accurate and culturally relevant information, mistrust in government services, concerns over vaccine safety, and past experiences of racism and abuse in healthcare settings; the socio-economic challenges of living in rural or remote areas with limited access to vaccination sites further hindered their ability to adopt preventive measures [8, 21, 30, 31].

The aim of this study is to evaluate COVID-19 vaccine uptake among Indigenous language speakers in Mexico, identify the barriers to accessing vaccines, and analyze how these barriers correlate with vaccine hesitancy. Using data from the latest national health survey, ENSANUT 2022, we seek to understand the vaccination disparities faced by Indigenous populations.

## Methods

### Data source

The data for this study was obtained from ENSANUT Continua 2022, a nationally representative survey conducted in Mexico between August and November 2022 [32]. The survey employed a cross-sectional stratified, multistage area probabilistic design, allowing for representation across different strata, including various ethnic, demographic, and regional groups. The stratification process involved dividing the national territory into primary sampling units (PSUs), with subsequent stages of selection ensuring a comprehensive sample [33]. Data collection in Indigenous languages is done using Indigenous language speakers or translators. More details about the general methods used for the survey can be found in the ENSANUT website. We included all participants included in ENSANUT 2022 that were over 5, since children under five were not eligible for the vaccine at the time of the survey.

### Outcome measurement

The COVID-19 vaccine status of participants was self-reported. Those who reported having received at least one dose of any COVID-19 vaccine were considered vaccinated. Among the unvaccinated individuals, the reasons for not getting vaccinated were categorized into three groups: 1) Access barriers, including factors like ineligibility, unavailability of vaccines, distant vaccination sites, long waiting lines, work commitments, disability, lack of accompaniment, time constraints, and illness; 2) belief barriers, which encompassed beliefs that the vaccine is ineffective, causes adverse effects on health, denial of the existence of COVID-19, preference to wait and see, distrust in the system and/or government, and fear of the vaccine; and 3) other reasons.

### Measurement of exposure

The primary exposure variable in our study was the self-reported ability to speak an indigenous language. This criterion is employed by ENSANUT to identify Indigenous participants. However, recognizing that not all indigenous individuals speak an indigenous language, we did not extend our findings to represent the entire indigenous population.

### Covariates

To ensure the integrity of our model, we selected covariates that avoided multicollinearity and aligned with established research. Relevant variables that had a high Variance Inflation Factor, including variables for socioeconomic status (SES) [household income and household characteristics] were omitted from the analysis. Rurality was classified according to the number of inhabitants as rural (less than 2,500 inhabitants) and urban (2,500 inhabitants or more). We dichotomized the variable representing insurance coverage, participants were categorized as ’yes’ if they had any form of insurance coverage (IMSS, ISSTE, private or other). Age was treated as a continuous variable. Educational attainment was categorized into five groups: no formal education, completion of preschool, primary education (1st-6th grade), secondary school (7th-9th grade), and preparatory school or college and technical college or higher education. Previous COVID-19 infection was self-reported by the survey respondents who were key-household members.

### Statistical analysis

We conducted all statistical analyses using StataIC V.16.0.809. The ’svy’ command was utilized to account for the survey design, and post-stratification weights were applied to adjust for discrepancies in the sample relative to the broader population. These weights correct for potential non-response biases and other survey design effects, ensuring that our findings accurately reflect the prevalence of vaccination among indigenous groups [34]. Baseline characteristics of participants who spoke an Indigenous language—and those who did not—were presented using percentages for categorical variables, and means with standard deviations for continuous variables. We calculated differences between the groups using Chi-square and Kruskal-Wallis tests.

For the assessment of COVID-19 vaccine uptake we used logistic regression to estimate odds ratios (ORs) with 95% confidence intervals (CIs) in unadjusted and adjusted models. Speaking an Indigenous language was the main predictor, and the primary outcome was having received at least one COVID-19 vaccine. We adjusted the model for potential confounders, including age, sex, educational attainment, and previous COVID-19 infection. To select confounders for the model, we used Variance Inflation Factor (VIF) tests to check for multicollinearity.

Lastly, we performed a logistic regression analysis to estimate ORs with 95% CIs for belief and access barriers, individually, among unvaccinated participants. For both analyses, the main predictor remained speaking an Indigenous language, and the primary outcomes were the citation of a belief barrier or access barrier (as described earlier) for not wanting a COVID-19 vaccine. We adjusted the model for possible confounders, including age, sex, and educational attainment.

### Ethics statement

This work is exempt from IRB (Institutional Review Board) approval since it involves secondary data analysis of a publicly available national survey. We were not involved in the data collection process, and all the data has been de-identified, making it impossible to link any record to a particular individual.

## Results

We included 34,051 participants in the study, out of which 1,793 individuals (5.23%) reported speaking an Indigenous language (Table 1). The average age of all participants was 35.8 years. Those who spoke an Indigenous language were, on average, older (40.9 years) compared to participants who did not speak an Indigenous language (35.6 years, p<0.005). Regarding gender distribution, no significant difference was observed between the two groups. In terms of educational attainment, participants who spoke an Indigenous language were more likely to have no formal education, 14%, compared to only 4% of non-Indigenous language speakers (p<0.005). Moreover, participants who spoke an Indigenous language were more likely to have completed fewer years of education compared to non-Indigenous language speakers. Indigenous language speakers were more likely to live in rural areas with less than 2500 people (p<0.005) and were less likely to have any type of medical insurance coverage (p<0.005).

**Table 1:** Characteristics and COVID vaccination history of respondents of ENSANUT Continua 2022, Mexico included in this study (N = 34,051).

	Total	Speaks an Indigenous language		P values
Characteristic	(n = 34,051) N (%)	No (n = 32,258) N (%)	Yes (n = 1793) N (%)	
**Age, years** ^**$**^	35.84 (21)	35.6 (21)	40.9 (21.3)	<0.005*
**Female sex**	17,987 (53%)	17,023 (53%)	964 (54%)	0.44
**Completed School level**				
None	1,473 (4%)	1,221 (4%)	252 (14%)	<0.005*
Preschool	959 (3%)	925 (3%)	34 (2%)	1.00
Primary School (1-6^th^)	10,264 (30%)	9,442 (29%)	823 (45%)	<0.005*
Secondary School (7-9^th^)	9,508 (28%)	9,088 (28%)	420 (23%)	<0.005*
Preparatory School (10^th^–12^th^)	6,162 (18%)	5,993 (19%)	169 (9%)	<0.005*
College, Technical College or more	5,684 (17%)	5,589 (17%)	95 (5%)	<0.005*
**Rural**	8,022 (24%)	7,024 (22%)	998 (57%)	<0.005*
**Uninsured**	16,443 (48%)	16,199 (50%)	1,409 (79%)	<0.005*
**Vaccinated against COVID-19 with at least one dose**	27,468 (80%)	26,339 (82%)	1,129 (63%)	<0.005*
**Previous COVID-19 infection**				
Yes	5,900 (17%)	5,756 (17%)	124 (8%)	<0.005*
No	27,759 (82%)	26,115 (81%)	1,664 (92%)	<0.005*
Not sure	392 (1%)	387 (1%)	5 (0%)	<0.005*

^$^Mean and SD

* for p < 0.05

Participants who spoke an Indigenous language had a significantly lower percentage (8%) of reporting a previous COVID-19 infection compared to those who did not speak an Indigenous language (17%) (p<0.005). Regarding COVID-19 vaccination, 80% of all participants had been vaccinated against COVID-19 with at least one dose. Participants who spoke an Indigenous language had a significantly lower vaccination rate (63%) compared to those who did not speak an Indigenous language (82%) (p<0.005).

Participants who reported speaking an Indigenous language were found to be 59% less likely to be vaccinated against COVID-19 compared to those who didn’t speak an Indigenous language (OR 0.41, 95% CI 0.27–0.62) even when adjusted for age, sex, and educational attainment, rurality and insurance status (Table 2). In both, the adjusted and unadjusted models, age appeared to be a significant factor. As age increases, the odds of being vaccinated also increased (Adjusted Model: OR 1.02, 95% CI 1.02–1.03). Women had significantly higher odds of being vaccinated compared to men (Adjusted Model: OR 1.22, 95% CI 1.12–1.32). Individuals with levels of education of primary school or above had significantly higher odds of being vaccinated compared to those with no formal education. The odds ratios increased as the level of education increased, with the highest odds observed for those with college, technical college, or higher education (Adjusted Model: OR 8.17, 95% CI 6.21–10.75). Living in a rural community was not a statistically significant predictor of being vaccinated. And those that didn’t have any health insurance had lower odds of being vaccinated (Adjusted Model: OR 0.54, 95% CI 0.49–0.61).

**Table 2:** Unadjusted and adjusted logistic regression models for the odds ratio of having at least a COVID-19 vaccine of respondents of ENSANUT Continua 2022, Mexico (N = 34,051).

	Unadjusted		Adjusted Model 1	
Characteristic	Odds Ratio (95% CI)	P value	Odds Ratio (95% CI)	P value
**Speaking an Indigenous language**	0.28 (0.17–0.46)	<0.005*	0.41 (0.27–0.62)	<0.005*
**Age**	1.03 (1.02–1.03)	<0.005*	1.02 (1.02–1.03)	<0.005*
**Sex**				
Male	Ref.		Ref.	
Female	1.21 (1.13–1.30)	<0.005*	1.22 (1.12–1.32)	<0.005*
**Completed school year**				
None	Ref.		Ref.	
Preschool	0.34 (0.26–0.44)	<0.005*	1.03 (0.75–1.41)	0.82
Primary School (1-6^th^)	0.98 (0.82–1.17)	0.86	1.65 (1.37–1.99)	<0.005*
Secondary School (7-9^th^)	1.90 (1.50–2.40)	<0.005*	2.97 (2.39–3.69)	<0.005*
Preparatory School 10^th^–12^th^)	3.62 (2.83–4.65)	<0.005*	5.55 (4.41–6.98)	<0.005*
College, Technical College or more	7.04 (5.41–9.17)	<0.005*	8.17 (6.21–10.75)	<0.005*
Rural	0.52 (0.42–0.65)	<0.005*	0.87 (0.72–1.06)	0.181
Uninsured	0.38 (0.33–0.43)	<0.005*	0.54 (0.49–0.61)	<0.005*

* for p < 0.05

With respect to the reasons for declining the COVID-19 vaccine (Table 3), out of the 6,582 unvaccinated participants, 2,645 individuals (40%) indicated an access-related obstacle, while 3,402 participants (52%) expressed a barrier related to their beliefs. Additionally, 535 respondents (8%) cited various other reasons for not receiving the vaccine. Participants who did not speak an Indigenous language reported a higher frequency of access barriers compared to those who spoke an Indigenous language, 2,438 (41%) and 207 (31%) respectively. Conversely, participants who spoke an Indigenous language were more likely to attributing their decision to a belief barrier, 414 (62%) participants, compared to 2,988 (51%) among those who did not speak an Indigenous language.

**Table 3:** Reasons for not getting a COVID-19 vaccine among unvaccinated (N = 6,582) participants of ENSANUT Continua 2022, Mexico.

Reason for not getting the COVID-19 vaccine	Total (n = 6,582)	Indigenous language speakers		P-value
		No (n = 5,918)	Yes (n = 664)	
The vaccine hasn’t arrived where we live	992 (15%)	920 (16%)	72 (11%)	<0.005*
It was very far for me	66 (1%)	48 (1%)	18 (3%)	<0.005*
The line was very long	222 (3%)	208 (4%)	14 (2%)	0.07
They didn’t let me leave my job	119 (2%)	111 (2%)	8 (1%)	0.28
I have a disability	32 (0.5%)	32 (0.5%)	0	0.11
I didn’t have anyone to accompany me	133 (2%)	125 (2%)	8 (1%)	0.15
Didn’t have time	682 (10%)	624 (10%)	58 (9%)	0.17
Was sick or due to some illness	399 (6%)	370 (6%)	29 (4%)	0.06
I believe the vaccine is ineffective	651 (10%)	551 (9%)	100 (15%)	<0.005*
I believe the vaccine has adverse effects/negative consequences for my health	994 (15%)	866 (15%)	128 (19%)	<0.005*
COVID is not a problem, it doesn’t exist	154 (2%)	134 (2%)	20 (3%)	0.28
I prefer to wait and see how things progress	168 (3%)	161 (3%)	7 (1%)	0.01 *
I don’t trust the system, the government	247 (4%)	229 (4%)	18 (3%)	0.17
Out of fear	1188 (18%)	1,047 (18%)	141 (21%)	0.03*
Other	535 (8%)	492 (8%)	43 (7%)	0.12

Reasons shaded in grey are belief barriers while the ones above were categorized as access barriers.

* for p < 0.05

Lastly, our analysis of unvaccinated individuals (N = 6,582) found that those who spoke an Indigenous language were more likely to report not being vaccinated due to negative beliefs about the vaccine or fear (OR 1.82, 95% CI 1.11–3.00) (Table 4). Among the confounders, higher levels of education were also associated with belief barriers, but this trend declined among those with a college or technical college degree (OR 1.24, 95% CI 0.79–1.95). Notably, among the unvaccinated, those who spoke an Indigenous language were less likely to report access barriers as reasons for not getting the vaccine compared to those who didn’t speak an Indigenous language in a model adjusted for age, sex, and educational attainment (OR 0.62, CI 95% 0.42–0.91) (S1 Table).

**Table 4:** Unadjusted and adjusted logistic regression models. Not getting vaccinated because of a belief barrier (fear, “it doesn’t work”, COVID isn’t real, etc.) among unvaccinated respondents of ENSANUT Continua 2022, México (N = 6,582).

	Unadjusted		Adjusted Model 1	
Characteristic	Odds Ratio (95% CI)	P value	Odds Ratio (95% CI)	P value
**Speaking an Indigenous language**	1.77 (1.19–2.63)	<0.005*	1.82 (1.11–3.00)	<0.005*
**Age**	1.03 (1.02–1.03)	<0.005*	1.02 (0.01–1.02)	<0.005*
**Sex**				
Male	Ref.		Ref.	
Female	0.98 (0.87–1.10)	0.75	0.98 (0.87–1.11)	<0.005*
**Completed school year**				
None	Ref.		Ref.	
Preschool	0.13 (0.09–0.19)	<0.005*	0.38 (0.24–0.60)	<0.005*
Primary School (1–6^th^)	0.56 (0.42–0.74)	<0.005*	0.99 (0.72–1.37)	0.99
Secondary School (7–9^th^)	0.91 (0.67–1.24)	0.053	1.58 (1.13–2.20)	<0.005*
Preparatory School (10^th^–12^th^)	1.02 (0.74–1.41)	0.569	1.74 (1.25–2.43)	<0.005*
College, Technical College or more	0.90 (0.58–1.41)	0.152	1.24 (0.79–1.95)	0.33

* for p < 0.05

## Discussion

In this study, we utilized publicly available data from Mexico’s nationally representative survey, ENSANUT Continua 2022, to examine COVID-19 vaccine uptake among different linguistic communities. The main variables that informed our study were: 1) having had at least one COVID-19 vaccine and 2) reason for not getting the vaccine. These two outcomes were then divided into access and belief barriers. We used logistic regression to analyze vaccine uptake and belief and access barriers among unvaccinated participants, adjusting for potential confounders. Our analysis of unvaccinated individuals found that those who spoke an Indigenous language were more likely to report not being vaccinated due to negative beliefs about the vaccine or fear. We also found that they were less likely to face access barriers to get the vaccine compared to those who didn’t speak an Indigenous language, challenging the common idea that Indigenous people are not vaccinated mostly because of a lack of access to healthcare.

Our main results, indicating that individuals speaking an Indigenous language in Mexico were less likely to be vaccinated against COVID-19, are consistent with the broader context of the pandemic’s impact on disadvantaged populations worldwide. Our findings align with studies conducted in other countries, which have also reported lower rates of COVID-19 vaccination in Indigenous communities. In Canada, for example, rates of vaccination among First Nations, Inuit, and Métis in Toronto and London were lower than in the general population [35]. Similarly, in Guatemala, vaccination rates have been low in high-risk Indigenous populations, and municipalities with a higher proportion of Indigenous people have experienced lower vaccine rates [36, 37]. In Colombia, one year after the initiation of COVID-19 vaccination, only 36.7% of the Indigenous population had been vaccinated (WHO), and in Brazil, vaccination coverage was 26% lower among Indigenous people when compared to the overall population [38, 39].

One potential limitation of our study is that vaccine status and perceived barriers were self-reported, which may introduce recall or social desirability bias. However, we mitigated this potential bias by using a large nationally representative survey, ENSANUT Continua 2022, which included diverse participants from various regions in Mexico. Also, we assumed that any bias in self-reporting would be equal in both groups (Indigenous language speakers and non-Indigenous language speakers), thus maintaining the internal consistency of our findings. Additionally, both vaccine status and reasons for not being vaccinated were coded by in-home interviewers, which may lead to some misclassification or coding errors. There could also be a risk of misinterpreting participants’ answers, potentially influencing the results.

The methodology employed by ENSANUT to ascertain Indigenous identity through language also presents a limitation to our study. We acknowledge that our findings may not be fully representative of the Indigenous population in Mexico, as there are individuals who identify as Indigenous but might not speak an Indigenous language. This discrepancy suggests the need for a broader approach to defining Indigenous status that goes beyond language use alone. We advocate for more inclusive criteria or alternative methodologies to capture the diversity of the indigenous population in Mexico more accurately. Lastly, the lack of direct partnership with Indigenous communities or Indigenous-led agencies in the study design is a notable limitation, potentially overlooking community-specific insights. Future research should prioritize collaboration with these groups to ensure culturally sensitive methodologies and more comprehensive understandings of the barriers to vaccination. This inclusive approach is crucial for the authenticity and relevance of our findings within the broader context of Indigenous health and vaccine equity.

Despite the limitations, our study offers several strengths that contribute to the validity and significance of the findings. By utilizing data from the nationally representative ENSANUT Continua 2022 survey, our results are generalizable to Indigenous language speakers throughout Mexico, providing valuable insights into the vaccine uptake and barriers among this understudied population. This study is the first of its kind to investigate COVID-19 vaccine uptake and reasons for not getting vaccinated specifically among Indigenous language speakers in Mexico. As such, our findings can inform targeted vaccine campaigns and future pandemic preparedness strategies aimed at addressing the needs of this disadvantaged population. By addressing the disparities identified in our study, public health interventions can be tailored to ensure equitable access to vaccination and better health outcomes for Indigenous communities.

The findings of this study have significant implications for public health, especially in the context of COVID-19, other vaccine preventable diseases and future pandemics. Given the severe impact of the COVID-19 pandemic on Indigenous communities, urgent attention is required to address existing inequities and improve health outcomes for these populations [12, 23, 30]. The study’s findings underscore the need for timely and inclusive vaccination campaigns that involve Indigenous communities in the planning and implementation process, emphasizing the importance of disseminating accurate and accessible information in Indigenous languages. Examples of such interventions have been successful in other countries, like Guatemala and India [36, 37]. Misinformation and lack of culturally appropriate information can hinder vaccination efforts. One-size-fits-all approaches may not effectively address the barriers faced by these populations, necessitating tailored strategies to improve vaccination rates.

Additionally, our findings may be contextualized within the framework of the ’3Cs’—confidence, complacency, and convenience—which are known factors contributing to vaccine hesitancy [40]. The belief barriers identified among Indigenous language speakers, including negative beliefs and fears, may reflect a lack of confidence in the vaccine’s safety and effectiveness. At the same time, the lower likelihood of facing access barriers suggests complacency may not be as significant a factor as initially thought. Future interventions should therefore address these aspects by building trust in healthcare services and emphasizing the severity of COVID-19 to counteract complacency and enhance convenience.

In Mexico, like many countries, the vaccination prioritization was primarily based on known risk factors like age and comorbidities, despite reports of higher mortality rates among Indigenous people in the country available as early as 2020, before the vaccine rollout. The failure to recognize and address these disparities in the early stages of the pandemic might have contributed to inequities in vaccine uptake interventions. Our study emphasizes the importance of considering social disadvantages in the prioritization of public health interventions, including vaccination campaigns. This evidence should ensure that resources and efforts are directed toward the most disadvantaged populations, such as Indigenous communities, who may face unique challenges in accessing vaccines. Furthermore, there is a pressing need for comprehensive data collection on race and ethnicity in vaccination and other public health data. ENSANUT currently does not collect information on race and ethnicity but rather languages spoken, therefore, our study highlights the role that race, and ethnicity play in vaccine uptake and barriers, underscoring the importance of having accurate data to guide public health policies.

In summary, this study sheds light on the disparities in COVID-19 vaccine uptake among Indigenous language speakers in Mexico. The findings indicate that this population is less likely to be vaccinated and more likely to cite negative beliefs and fears as reasons for not getting vaccinated. These results align with the broader context of the pandemic’s impact on disadvantaged populations worldwide and are consistent with studies conducted in other countries.

## Supporting information

S1 Checklist Inclusivity in global research questionnaire. (DOCX)

S1 Table Unadjusted and adjusted logistic regression models not getting vaccinated because of an access barrier among unvaccinated respondents of ENSANUT Continua 2022, México (N = 6,582). (DOCX)
