# Supplementary material for: COVID-19 vaccine uptake and barriers among Indigenous language speakers in Mexico: Results from a nationally representative survey
Source: PLOS Glob Public Health. 2024 Mar 28;4(3):e0002921. doi: 10.1371/journal.pgph.0002921 (PMC10977884; doi:10.1371/journal.pgph.0002921)
Supplement: S1 Table — (DOCX) [file pgph.0002921.s002.docx]

|  | | **Unadjusted** | | **Adjusted Model 1** | |
| --- | --- | --- | --- | --- | --- |
| **Characteristic** | **Odds Ratio**  **(95% CI)** | | **P value** | **Odds Ratio**  **(95% CI)** | **P value** |
| **Speaking an Indigenous language** | 0.68 (0.56-0.81) | | <0.005* | 0.62 (0.42-0.91) | <0.005* |
| **Age** | 0.96 (0.96-0.97) | | <0.005* | 0.97 (0.96-0.97) | <0.005* |
| **Sex** |  | |  |  |  |
| Male | Ref. | |  | Ref. |  |
| Female | 0.93 (0.84-1.03) | | 0.18 | 0.91 (0.96-1.01) | 0.10 |
| **Completed school year** |  | |  |  |  |
| None | Ref. | |  | Ref. |  |
| Preschool | 5.59 (4.18-7.46) | | <0.005* | 1.69 (1.22-2.34) | <0.005* |
| Primary School (1-6^th^) | 1.84 (1.45-2.34) | | <0.005* | 0.91 (0.70-1.18) | 0.49 |
| Secondary School (7-9^th^) | 1.30 (1.01-1.66) | | 0.037 | 0.67 (0.51-0.88) | <0.005* |
| Preparatory School (10^th^–12^th^) | 1.08 (0.81-1.44) | | 0.577 | 0.58 (0.43-0.79) | <0.005* |
| College, Technical College or more | 1.43 (1.04-1.98) | | 0.152 | 0.99 (0.71-1.39) | 0.98 |
|  |  | |  |  |  |

### **Supplemental Material Table 1.** Unadjusted and adjusted logistic regression models Not getting vaccinated because of an access barrier among unvaccinated respondents of ENSANUT Continua 2022, México (N=6,582)

* for p < 0.05
